# Enhanced Arsenic Tolerance in *Triticum aestivum* Inoculated with Arsenic-Resistant and Plant Growth Promoter Microorganisms from a Heavy Metal-Polluted Soil

**DOI:** 10.3390/microorganisms7090348

**Published:** 2019-09-12

**Authors:** Javiera Soto, Javier Ortiz, Hector Herrera, Alejandra Fuentes, Leonardo Almonacid, Trevor C. Charles, César Arriagada

**Affiliations:** 1 Laboratorio de Biorremediación, Facultad de Ciencias Agropecuarias y Forestales, Universidad de La Frontera, Francisco Salazar, Temuco 01145, Chile; 2 Programa de Doctorado en Ciencias mención Biología Celular y Molecular, Universidad de La Frontera, Francisco Salazar, Temuco 01145, Chile; 3 Department of Biology, University of Waterloo, University Avenue West, Waterloo, ON N2L 3G1, Canada

**Keywords:** arsenic contamination, oxidative stress, plant growth promotion, soil microorganisms

## Abstract

In soils multi-contaminated with heavy metal and metalloids, the establishment of plant species is often hampered due to toxicity. This may be overcome through the inoculation of beneficial soil microorganisms. In this study, two arsenic-resistant bacterial isolates, classified as *Pseudomonas gessardii* and *Brevundimonas intermedia*, and two arsenic-resistant fungi, classified as *Fimetariella rabenhortii* and *Hormonema viticola*, were isolated from contaminated soil from the Puchuncaví valley (Chile). Their ability to produce indoleacetic acid and siderophores and mediate phosphate solubilization as plant growth-promoting properties were evaluated, as well as levels of arsenic resistance. A real time PCR applied to *Triticum aestivum* that grew in soil inoculated with the bacterial and fungal isolates was performed to observe differences in the relative expression of heavy metal stress defense genes. The minimum inhibitory concentration of the bacterial strains to arsenate was up to 7000 mg·L^−1^ and that of the fungal strains was up to 2500 mg·L^−1^. *P. gessardi* was able to produce siderophores and solubilize phosphate; meanwhile, *B. intermedia* and both fungi produced indoleacetic acid. Plant dry biomass was increased and the relative expression of plant metallothionein, superoxide dismutase, ascorbate peroxidase and phytochelatin synthase genes were overexpressed when *P. gessardii* plus *B. intermedia* were inoculated.

## 1. Introduction

Arsenic (As) is a toxic metalloid that is distributed extensively in the environment due to natural geochemical processes and anthropogenic activities. Environmental As can derive from several natural sources such as volcanoes, marine sedimentary rocks and fossil fuels, although mainly from anthropogenic activities, including metal smelting, mining, agricultural chemicals (pesticides), wood preservatives and industrial activities [1,2]. Soil contamination with As has become a serious environmental and human hazard. The US Environmental Protection Agency [3] lists As as a carcinogenic substance. It exists in the environment mainly as oxyanions of trivalent arsenite As(III) or pentavalent arsenate As(V), with As(III) being more toxic than As(V) in biological systems [4]. There is a structural similarity between arsenate and inorganic phosphate, so it can enter the cell easily through the same transport system as phosphate. This mimicry allows arsenate to alter phosphate metabolism [5,6].

Arsenic is toxic to plants because it interferes with their metabolism, causing disorders at various levels of organization, inhibiting plant growth [5]. Arsenic is accumulated predominantly in the root system, where the metalloid inhibits biomass accumulation [7,8,9]. At elevated concentrations, As hampers critical metabolic processes, which can lead to plant death [10]. Several plants have developed mechanisms to sequester As in the roots; however, As can be translocated to other plant tissues, altering growth and yield, causing discoloration and plasmolysis of the root, wilting and necrosis of the apex and contour of the leaves, inhibition of chlorophyll biosynthesis, and a decrease in photosynthetic capacity [11,12].

Plants have developed a series of strategies to withstand the toxicity of arsenic, such as i) the reduction of As(V) to As(III); ii) As(III) chelation by glutathione; iii) chelation by phytochelatins (PC); iv) the sequestration of As complexes in vacuoles [13,14]. Within the plant cell, As(V) interferes with oxidative phosphorylation and the synthesis of ATP in the mitochondria by replacing phosphate [15], whereas As(III) binds to sulfhydryl groups, with the consequent detrimental effects on protein function [13,16]. Likewise, As toxicity is caused by the effects of oxidative stress, although the exact molecular mechanisms affected are still not clear [17].

Recent studies have shown that microorganisms isolated from heavy metal-polluted soils can effectively promote plant growth [18,19,20]. When considering approaches to alter the mobilization of heavy metals, there are several advantages of using microorganisms instead of chemical methods, since metabolites produced by microorganisms are biodegradable, less toxic and can be produced in situ [21]. In addition, microorganisms associated with plants are able to produce growth-regulating substances such as siderophores and indoleacetic acid, and mediate phosphate solubilization, which increases the growth of plants in a soil contaminated with metals [22,23,24]. We hypothesized that the inoculation of bacterial and fungal strains isolated from heavy metal-polluted soils promotes plant growth when they are subjected to high concentrations of arsenic and generate greater tolerance to oxidative stress. Therefore, the aims of this study were to characterize the production of some secondary metabolites, such as indoleacetic acid, phosphate solubilization and siderophores, from arsenic-tolerant bacteria and fungi isolated from the rhizosphere of metallophyte plants and to evaluate the effects of these microorganisms on reducing oxidative stress and promotion of the plant growth of wheat plants grown in a metal-polluted soil.

## 2. Materials and Methods

### 2.1. Study Site

The soils used in this study were collected from a rhizospheric area, i.e., the closest region of the soil that is directly influenced by root exudates of *Oenothera picensis* plants located in the Puchuncaví Valley in the coastal area of central Chile (V Region), 1.5 km southeast of the Ventanas copper smelter (32°46′ 30″ S; 71°28′17″ W) that has been subjected to large quantities of gaseous and metal-rich particulate pollution from the copper smelter since 1964 [25]. The soil is classified as an Entisol and has a pH of 5.54 and 2.41% organic matter [26]. Characteristics of the soils used are described in Table 1. The pH of soil samples was determined in a 1:2.5 (*v*/*v*) suspension of soil in H_2_O. Electrical conductivity (CE) was measured on samples at a ratio of 1:5 (sample: water) and shaken for 30 min. Organic matter was determined by wet digestion using the Walkley and Black [27] method. Total N concentration was obtained using the Kjeldahl method. Olsen-P was measured using the Olsen P test, in which inorganic P is extracted from soil with 0.5 M NaHCO_3_ at pH 8.5 [28]. Available K and total concentrations of heavy metals (Cu, Zn, Cd and Pb) were determined as described by Mingorance [29].

### 2.2. Isolation of Bacteria and Fungi and Their Resistance to Arsenic

Bacteria and fungi were isolated from the rhizospheric heavy metal-polluted soil samples described above. Bacteria were isolated by adding 1 g of homogenized soil to 10 mL of sterile distilled water, and shaking at 100 rpm for 5 min. The mixture was serially diluted, and 0.1 mL aliquots were plated onto LB (Luria-Bertani) agar plates (10 g tryptone, 10 g NaCl, 5 g yeast extract, 12 g agar per liter) and incubated at 27 °C for 48 h. After growth, a number of different colonies were purified. The fungi were isolated according to the method described by Bissett and Widden [30]. The isolated fungi were kept in plates with potato dextrose agar (PDA) culture medium at 4 °C with periodic subculture. Pure cultures of a total of 10 bacteria and 8 fungi were obtained, based on their different macroscopic characteristics (colonial morphology, color, texture, shape, and diameter).

The isolated bacterial and fungal strains were tested for their tolerance to As(V) as sodium arsenate (Na_2_HAsO_4_·7H_2_O) and sodium (meta)arsenite (NaAsO_2_) (Sigma-Aldrich, St. Louis, MS, USA). LB broth and PDA were used for As(V) and As(III) experiments. Bacterial strains were cultured in modified LB broth that was supplemented with As(V) and As(III). The concentrations used ranged from 500 to 8000 which increased 500 mg·L^−1^ each time. The strains were cultured in an orbital shaker at 27 °C for 72 h, and growth was monitored by measuring their OD600. Fungal strains were placed in the center of Petri dishes containing PDA plus As(V) and As(III), the concentration ranged between 500 and 3000 mg·L^−1^, which increased 500 mg·L^−1^ each time, and were incubated for 21 days at 27 °C. Fungal growth was measured in mm. Among the microorganisms tested, two bacteria (designated as strains B4 and B10) and two fungi (designated as strains V7 and V8) were finally chosen for the following experiments for their ability to resist high concentrations of As(V) and As(III).

### 2.3. Molecular Characterization

Bacterial DNA extraction was made using the UltraClean^®^ Microbial DNA Isolation Kit (MO-BIO Laboratories, Carlsbad, CA, USA) according to the manufacturer’s instructions. Molecular identification of bacteria was performed by amplification of 16S rDNA gene through PCR according to Banerjee, et al. [31] using 27F (5′-AGAGTTTGATCCTGGCTCAG-3′) and 1492R (5′-TACGGTTACCTTGTTAC GACTT-3′) primers. DNA extraction of the fungi was done using the DNeasy Plant Mini Kit (QIAGEN, Hilden, Germany) and subsequent PCR amplification of the 18S rDNA with the universal primers ITS1 and ITS4 [32] following the PCR conditions described in Herrera, et al. [33]. DNA sequencing was performed by Macrogen (Seoul, Korea). A BLAST (Basic Local Alignment Search Tool) search against the GenBank database (http://www.ncbi.nlm.nih.gov/) was conducted to find the closest known sequences of microorganisms.

### 2.4. Screening of Potential Plant Growth-Promoting Traits

Production of siderophores was determined according to Milagres, et al. [34]. Chrome azurol S (CAS) (Sigma-Aldrich, St. Louis, MO, USA) was prepared according to Schwyn and Neilands [35]. A volume of 20 mL of LB agar or PDA medium was dispensed into Petri dishes. After that, the solidified medium was cut into halves, one of which was replaced by CAS-blue agar. The bacteria and fungi were inoculated in the center of the two solidified media and incubated at 27 °C in the dark for 5 days. The CAS reaction rate was determined by the color-change front in the CAS-blue agar, expressed as (+) when the color change was present, and as (−) when it was not.

To measure indoleacetic acid production (IAA), bacterial and fungal isolates were transferred to sucrose minimal salt (SMS) medium (10 g·L^−1^ sucrose, 2 g·L^−1^ K_2_HPO_4_, 1 g·L^−1^ (NH_4_)_2_SO_4_, 0.5 g·L^−1^ MgSO_4_, 0.5 g·L^−1^ yeast extract, 0.5 g·L^−1^ CaCO_3_, 0.1 g·L^−1^ NaCl, pH 7.2) and 1/7 PDB, respectively, both supplemented with 500 mg·mL^−1^ l-tryptophan and 250 mg·L^−1^ of sodium arsenate. The cultures were incubated in an orbital shaker at 27 °C for 48 h (bacteria) and 7 d (fungi). The culture solution was centrifuged at 2000 *g* for 20 min, and a 0.5 mL portion of the supernatant was mixed with 2 mL of Salkowski’s reagent [36]. The absorbance of the mixture was measured at 530 nm after 25 min of reaction. Pure IAA was used as a standard, and the concentration of the extracts was calculated and expressed in µg L^−1^. To determine phosphate solubilization, the bacterial and fungal strains were inoculated on Pikovskaya agar (HiMedia Laboratories, Dindori, India) medium and incubated for 120 h at 28 °C. Their phosphorus solubilizing activity was then determined by measuring the size of the halo formed by the solubilization of insoluble phosphate [37]. The results are shown as positive (+) or negative (−) solubilization.

### 2.5. Establishment of Plants and Inoculations

The experiments were carried out using *Triticum aestivum* var PANTERA-INIA due to its rapid growth, good performance in the heavy metal-polluted soil used, and the availability of extensive molecular information in the databases. Seeds were sterilized with 0.05% NaClO for 15 min under agitation and rinsed with abundant sterile distilled water; they were sown in vermiculite and left in darkness until germination. After ten days, three seedlings were transplanted to 300 cc pots with Puchuncaví Valley soil, sterilized in an autoclave for three consecutive days, supplemented with 40 mL of 0 and 300 mg·L^−1^ of As(V) which was previously incubated for 14 days.

The bacterial and fungal strains were inoculated to the soil individually and together (the 2 bacteria mixed, and the 2 fungi mixed). A volume of 10 mL of a solution of distilled water with each bacterium was taken at an optical density of 0.5 measured at 600 nm. For the fungi, a Petri dish with fungal hyphae covering the total surface was crushed and diluted in 40 mL of sterile distilled water. For each pot, 10 mL of each solution was inoculated. Plants were cultivated in a greenhouse for 14 days and then transferred to the growth chamber with light provided by Sylvania (Middleton, MA, USA) incandescent lamps—cold white light (400 E m^−2^ s^−1^, 500–700 nm), with a cycle of 16/8 h day/night at 24/16 °C and 50% relative humidity. Each treatment consisted of 12 replicates and the plants were harvested after 20 days. Shoot and root dry biomass was determined using a forced air stove (65 °C for 72 h), weighed on a digital scale and expressed in grams. The total As content in *T. aestivum* was determined as described by Mingorance [29]. Plants were frozen in liquid nitrogen and stored at −80 °C for further analyses.

The experiment design consisted of: (i) control, (ii) the inoculation of bacteria ((1) *P.gessardii*, (2) *B. intermedia*, and (3) *P.gessardii* plus *B. intermedia*) and fungi ((4) *F. rabenhortii,* (5) *H. viticola,* and (6) *F. rabenhortii* plus *H. viticola*) to *Triticum aestivum* plants (iii) growing under 0 and 300 mg K^−1^ of As. There were 14 treatments with 12 biological replicates.

### 2.6. Soil Biochemical Properties

To observe some possible soil biochemical changes, we studied acid phosphatase activity and it was measured using *p*-nitrophenyl phosphate (PNPP) (Sigma-Aldrich, St. Louis, MS, USA) as a substrate, according to the method described by Gianfreda, et al. [38]. The β-glucosidase activity was determined by detection of *p*-nitrophenol (PNP) released from *p*-nitrophenyl-β-d-glucopyranoside (PNG) (Sigma-Aldrich, St. Louis, MS, USA). In both assays, the amount of PNP formed was determined by spectrophotometry at 398 nm [39]. The hydrolysis of fluorescein diacetate (FDA) as an indicator of the total microbial activity of soil was determined according to Adam and Duncan [40] and expressed as μg fluorescein released per g of dry soil. The final concentration of FDA was measured as the absorbance at 490 nm.

### 2.7. Real-Time PCR

Total RNA was isolated from 100 mg of leaves using the RNA-Solv reagent (E.Z.N.A.^®^, Omega Bio-tek, Norcross, GA, USA) following the manufacturer’s instructions where the centrifugation temperature was modified to 4 °C. The samples were treated with RNase-free DNase I Set (E.Z.N.A.^®^) and purified using HiBind RNA mini Columns (E.Z.N.A.^®^). The concentration and purity of the extracted RNA were determined using an Infinite^®^ 200 NanoQuant Plate™ (TECAN, Seestrasse, Switzerland). RNA quality was verified by denaturing agarose gel visualization. Primers for real-time PCR analyses were designed from mRNA sequences of *Triticum aestivum* stored in the NCBI database (http://www.ncbi.nlm.nih.gov/). Each primer was tested in silico in an attempt to avoid dimer formation. Target genes (superoxide dismutase, ascorbate peroxidase, metallothionein, and phytochelatin) are related to heavy metal stress defense genes. The genes *GAPDH*, *α-TUB* and *EF1α* were chosen as candidates for reference genes. Descriptions of the designed primers are shown in Table 2.

The synthesis of the first strand of cDNA was obtained from 1 μg total RNA using the AffinityScript cDNA Synthesis Kit (Agilent Technologies, Santa Clara, CA, USA) following the manufacturer’s instructions. The qPCR reaction was performed in a final volume of 20 μL, using 2 μL of pre-diluted cDNA 1:10 and a mixture of 0.625 μL of each primer (10 μM), 10 μL of PowerUp™ SYBR™ Green Master Mix (Applied Biosystems™, Foster City, CA, USA) and 7.5 μL of nuclease-free water. For the negative control, the same reaction without template was used. Real-time PCR was carried out in a StepOnePlus Thermocycler (Applied Biosystems™, Foster City, CA, USA) using the following thermal profile: 10 min at 95 °C and 40 cycles of 15 s at 95 °C, 15 s at 60 °C, and 15 s at 72 °C. Data were processed using the Gene Expression Analysis for iCycler iQ real-time PCR Detection System software (BioRad Laboratories, Hercules, CA, USA).

### 2.8. Statistical Analysis

Statistical analyses were carried out with StatSoft Inc. (2004) STATISTICA (data analysis software system), version 7 (www.statsoft.com) (Tulsa, OK, USA). Differences between treatments were evaluated using a factorial analysis of variance (ANOVA). The Tukey test was used to observe differences between groups. Statistical significance was determined at *p* < 0.05.

## 3. Results

### 3.1. Bacteria and Fungi Identification and Arsenic Resistance

The bacterial isolates B4 and B10 showed 100% identity with *Pseudomonas gessardii* (Accession Number MH398505.1) and 100% identity with *Brevundimonas intermedia* (Accession number KR811205.1), respectively. The fungal isolates V7 and V8 showed 100% identity with *Fimetariella rabenhortii* (Accession Number HQ406808) and *Hormonema viticola* (Accession number NR137620.1), respectively.

Minimum inhibition concentrations (MICs) of fungal and bacterial strains to As are shown in Table 3. Both bacterial strains showed resistance to high concentrations of sodium arsenate. *P. gessardii* and *B. intermedia* were able to grow in LB medium supplemented with 7000 and 6000 mg·L^−1^ of As, respectively. In the case of both fungal strains, *F. rabenhortii* and *H. viticola* were able to grow in 2500 mg·L^−1^ of As.

### 3.2. Screening of Potential Plant Growth-Promoting Traits

The production of low molecular weight molecule, iron-chelating siderophores by the arsenic-resistant bacterial and fungal strains detected by the Chrome azurol S (CAS) assay are shown in Table 3. Only *P. gessardii* produced changes in medium coloration to orange, which is consistent with chemical testing for hydroxamate siderophores [41]. The bacteria and fungi isolates screened for IAA production showed that one bacterial strain and both fungi were able to utilize l-tryptophan as a precursor to producing IAA (Table 3), and the presence of arsenic had no negative effect on IAA production. *B. intermedia* produced 52.45 µg mL^−1^ and *P. gessardii* had no production. Meanwhile, *H. viticola* produced the highest IAA concentration (57.54 µg mL^−1^), and As supplementation did not affect IAA production by *F. rabenhortii* and *H. viticola*. Regarding phosphate solubilization, only *P. gessardii* was able to solubilize phosphate in solid culture (Table 3), showing a clear formation of a solubilization halo.

### 3.3. Shoot and Root Dry Biomass

**Figure 1:**
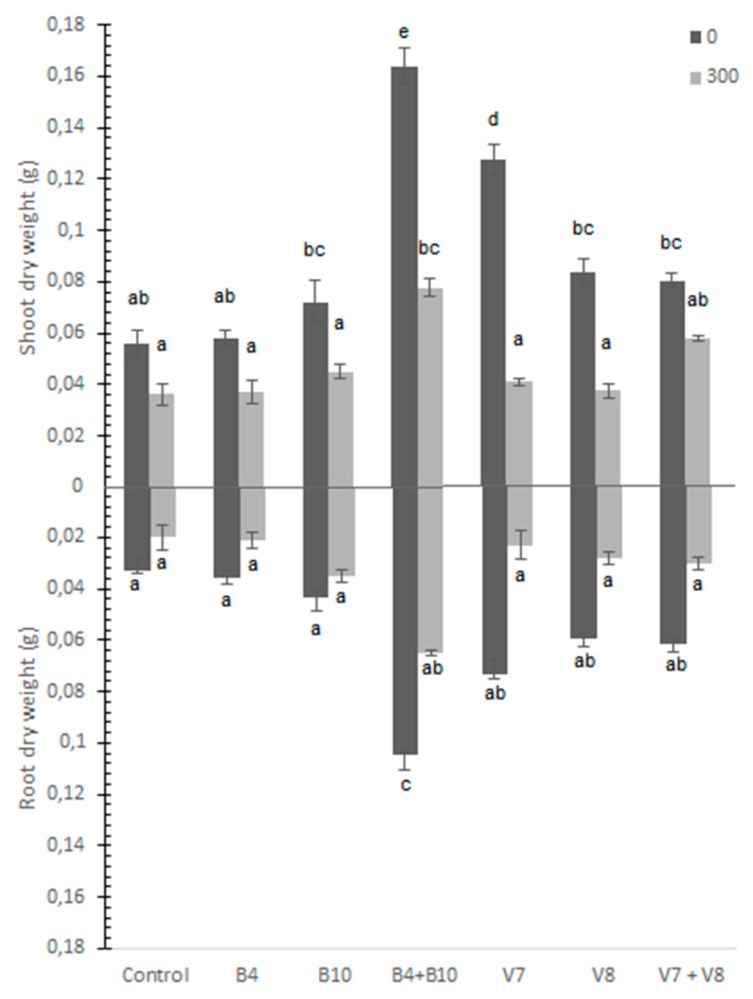
Shoot and root dry biomass of *Triticum aestivum* grown in a soil supplemented with 300 mg·Kg^−1^ of As and inoculated with *Pseudomonas gessardii* (B4), *B. intermedia* (B10), the mixture of both (B4 + B10), *F. rabenhortii* (V7), *H. viticola* (V8), and the mixture of both (V7 + V8). The standard deviation is indicated by different bars and letters show statistically significant differences (*p* < 0.05).

The plants that grew in the soil supplemented with As showed lower shoot and root dry biomass and decreased between 30% and 50% approximately (Figure 1); nevertheless, the treatment with the mixture of *P. gessardii* plus *B. intermedia* (B4 + B10) showed a significant increase compared to the control. Also, shoot dry weight increased with the inoculation of *F. rabenhortii* (V7) and there is a positive tendency with the inoculation of *B. intermedia* plus *P. gessardii* (B4 + B10) when As was not added to the soil. Arsenic content (Table A1) in the shoot and root of *Triticum aestivum* without the addition of As and the addition of 300 mg·Kg^−1^ of As showed no differences among the treatments.

### 3.4. Soil Enzyme Activities

Acid phosphatase activity, associated with phosphate solubilization, was substantially increased between 56% and 145% in treatments *B. intermedia*, *F. rabenhortii*, and *F. rabenhortii* plus *H. viticola* in soils without added As, and approximately 92% with the inoculation of *B. intermedia* and *H. viticola* in soils supplemented with As (Table 4). For β-glucosidase activity and fluorescein diacetate activity (FDA) hydrolysis, there were no significant differences between treatments. 

### 3.5. Relative Quantification of Gene Expression

**Figure 2:**
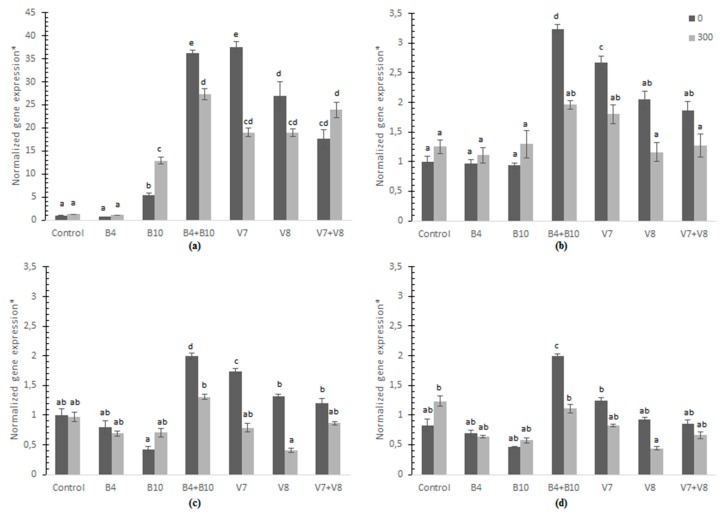
Relative expression of the metallothionein (*MT*) (**a**), superoxide dismutase (*Zn/Cu SOD*) (**b**), ascorbate peroxidase (*APX*) (**c**) and phytochelatin synthase (*PCS*) (**d**) genes in *T. aestivum* leaves grown in a soil supplemented with 300 mg·Kg^−1^ of As and inoculated with *P. gessardii* (B4), *B. intermedia* (B10), the mixture of both (B4 + B10), *F. rabenhortii* (V7), *H. viticola* (V8), the mixture of both (V7 + V8). The standard deviation is indicated by different bars and letters show statistically significant differences (*p* < 0.05). * Normalized to *GAPDH* and *α-TUB*.

The expression of genes involved in the stress response caused by As in wheat leaves was analyzed. The control treatment (without the addition of As) was defined as a calibrator and is expressed in the graphs with the value 1; therefore, all the treatments were compared with the control. Figure 2a shows the relative expression of the gene that codes for a metallothionein (*MT*), which showed a 5-fold increase when the plants were inoculated with *B. intermedia* (B10) and a 10-fold increase when As was added. Inoculation with *P. gessardii* plus *B. intermedia* (B4 + B10), and the fungus *F. rabenhortii* plus *H. viticola* (V7 + V8) showed up to a 35-fold increase. Increased expression was observed in all treatments, except with inoculation with *P. gessardii* (B4), when the soil was supplemented with 300 mg·Kg^−1^ of As. The relative expression of the superoxide dismutase (*Zn*/*Cu SOD*) and ascorbate peroxidase (*APX*) genes (Figure 2b,c) increased in plants inoculated with *P. gessardii* plus *B. intermedia* (B4 + B10) and the fungi *F. rabenhortii* (V7) without the As added.

The relative expression of the phytochelatin synthase (*PCS*) gene (Figure 2d) indicates a tendency to decrease when bacteria were inoculated separately in soils with or without the supplementation with As. As the inoculation of *P. gessardii* plus *B. intermedia* (B4 + B10) increased, the relative expression of *PCS* decreased compared to the control.

## 4. Discussion

Arsenic is toxic to bacteria and fungi, as well as to other life forms. The isolates *P. gessardii*, *B. intermedia* and the fungi *F. rabenhortii* and *H. viticola* can resist high concentrations of As, either as As(III) or As(V). This characteristic is likely associated with evolutionary adaptation to soil highly contaminated with a variety of heavy metals from which they were isolated, and these stressful conditions have led them to develop mechanisms to resist and tolerate restrictive edaphic conditions [42]. To cope with the As toxicity, As resistance (ars) genes can be found in the genome of nearly every bacterial species sequenced to date [43], and both, bacteria and fungi have the ability to tolerate As by several mechanisms including valence transformation, extra and intracellular precipitation, active uptake and biosorption [44]. Several studies have shown that the genera *Pseudomonas* [45,46] can reduce or oxidize arsenic and Tsubouchi and Kaneko [47] showed that *Brevundimonas* possess the ars operon that allow them to tolerate As by reduction or oxidation, among other processes. In regard to the fungi used in our study, there is no information about how arsenics metabolize.

Interactions between plants and microorganisms in the rhizosphere play a key role in growth of plants, and in nutrient uptake and transport [48]. Several studies have shown that plants’ adaptation to local environmental stress is closely related to the microorganisms that are in their surroundings [49]. Thereby, the evaluation of plant growth-promoting traits is of the utmost importance since these minimize toxic effects of abiotic and biotic stress including heavy metals in our study. Bacterial and fungal strains showed that *P. gessardii* produced siderophores and solubilized phosphate, and *B. intermedia*, *F. rabenhortii* and *H. viticola* were able to synthetize indoleacetic acid (IAA). It has been reported that As-resistant microorganisms belonging to diverse species possess plant growth-promoting traits, Das, et al. [50] and Xu, et al. [51] showed a variety of bacteria genera, such as *Brevundimonas*, *Pseudomonas*, *Comamona, Bacillus*, among others, that were able to produce siderophores, IAA and are capable of solubilizing phosphate.

Because these microorganisms were isolated from multicontaminated soil, they have the advantage of being adapted to this stressful condition and the production of IAA was not affected by the presence of As in the medium. In fact, this was increased in the case of *F. rabernhortii*. Carlos, et al. [52] showed that the production of IAA could show a 2-fold increase and this can happen with several other heavy metals. These characteristics are likely to play a key role in the adaptation of plants to a new environment [53] as a direct mechanism to promote tolerance and growth, because siderophores make iron available for plants, even under conditions of limited essential metal availability [54], and together with an effective inducer of plant growth such as IAA [55] will contribute to the adaptation of plants in heavy metal and As polluted soil [56].

In the present study, the growth of *T. aestivum* showed an inhibition of shoot and root dry weight due to the presence of a high level of As. In fact, this growth reduction induced by As is known to be due to the inactivation of enzymes by the binding of As with –SH protein groups with detrimental effects on protein function [16], oxidative stress, and by deterioration in other cell functions [57,58]. Although the high As the concentration added to the soil negatively affected plant growth, inoculation with *B. intermedia*, *H. viticola* and *F. rabenhortii* plus *H. viticola* had a tendency to show an improved positive effect on plant biomass when As was not added to the soil, and inoculation with *B. intermedia* plus *P. gessardii* and *F. rabenhortii* did increase plant growth in these conditions; it is important to note that the soil used in this experiment already had high concentrations of heavy metals (Table 1) and thus the microorganisms used could help in this type of condition but not with an excess of As. The inoculation of *B. intermedia* plus *P. gessardii* and *F. rabenhortii* increased the dry biomass of *T. aestivum* in the control soil. This joint effect can occur due to the metabolites, such as IAA, siderophores and phosphate solubilization that both microorganisms release and that can be complementary, having a synergistic effect [59]. IAA regulates growth and developmental processes [60]. Therefore, it directly intervenes in the increase in plant growth. Furthermore, *P. gessardii* showed an ability to solubilize phosphate and produce siderophores, likely self-regulating the iron in the soil and reducing the toxicity of arsenic (V) by increasing the iron supply to the plant [61]. In addition, the concentration of heavy metals and arsenic found in this soil interferes with the absorption of several nutrients such as Fe and P, thus inhibiting plant growth. In this context, microorganisms that have the ability to solubilize phosphate can provide soluble forms of P for plants and make an important contribution to the growth-promoting effect in plants [62]. Likewise, *B. intermedia* as well as both fungi produced a high concentration of IAA. This phytohormone directly stimulates plant cell elongation and cell division [63], which leads to a stimulation of plant growth and reduces the stress caused by soil pollution with heavy metals and arsenic.

The transcription of genes involved in the stress response by heavy metals in wheat leaves showed that the relative expression of the genes *MT*, *SOD*, *APX* and *PCS* was stimulated in the leaves when *P. gessardii* plus *B. intermedia* and in some cases *F. rabenhortii* were present. It is reported that the IAA released by rhizobacteria, in this case, *B. intermedia*, could directly promote the growth of roots by stimulating elongation of the plant cells or increasing cell division [64], which may enhance the root As absorption. Moreover, siderophore production by *P. gessardii* may mobilize As(V) in the soil in the process of taking up iron ions [65], which renders As more soluble and bioavailable to plants, which may explain the overexpression of these genes when *P. gessardii* plus *B. intermedia* where inoculated together. Even though it could be an increase in the exposure to inorganic As and the induction of reactive oxygen species (ROS), these microorganisms are stimulating the defenses against ROS by increasing the production of antioxidant enzymes and *MT* and *PCS*, and can be corroborated with the increase in dry biomass (Figure 1). Abercrombie, et al. [66] found in *Arabidopsis thaliana* that the *Zn*/*Cu SOD* class is at least doubled in the presence of As (V). Therefore, the enzymatic mechanisms effectively counteract the oxidative stress induced by As. Likewise, the variation in enzymatic antioxidant activities is related to the availability of nutrients in the soil, since some of these are metalloenzymes and their activities are determined by the availability of heavy metals [67].

## 5. Conclusions

Although not all the microorganisms studied had a beneficial effect for the plants, inoculation with the bacterium *P. gessardii* plus *B. intermedia* promoted growth in wheat plant and produced a higher antioxidant enzymatic response to the supplementation of the soil with As, showing the potential of the microorganisms isolated from polluted soil to contribute to the restoration of contaminated soils. In this order, further studies will be focused on understanding the nature of the mechanisms involved in the interactions between other plants and microorganisms and finding applications in the development of bioremediation strategies. Furthermore, the application of heavy metal-resistant and plant-beneficial bacteria and fungi can be considered as a tool for bioremediation strategies with great economic and ecological importance.

## Figures and Tables

**Table 1:** Chemical analysis of the soil sample from the Puchuncaví Valley.

**pH (Water)**	5.54 ± 0.13
**Electrical conductivity (CE) (dS L^−1^)**	0.1 ± 0.09
**Organic matter (%)**	2.41 ± 0.16
**N total (%)**	0.1 ± 0.02
**N available (mg** **·kg^−1^)**	28.7 ± 1.78
**P available (mg** **·kg^−1^)**	40.3 ± 3.47
**K interchangeable (mg** **·kg^−1^)**	210 ± 8.21
**Cu total (mg** **·kg^−1^)**	385 ± 14.53
**Zn total (mg** **·kg^−1^)**	183 ± 7.93
**Cd total (mg** **·kg^−1^)**	1.1 ± 0.07
**Pb total (mg** **·kg^−1^)**	135 ± 6.91
**As total (mg** **·kg^−1^)**	52 ± 3.65

**Table 2:** Set of primers used for real-time PCR.

Gene	Function		Primers 5′–3′	Amplicon Length (pb)
*GAPDH*	Glyceraldehyde-3-phosphate dehydrogenase	F	CCGTGTTCCCACTGTTGATGTT	192
		R	GCATCAAAGATGCTGGACCTGT	
*α-TUB*	Alpha-tubulin	F	CTGACAGCTTCCCTGAGGTTTGAT	179
		R	TCAAAGGCGCTGTTGGTGATCT	
*PCS*	Phytochelatin synthase	F	GCTATGTGGTAGTTGCTCGTCTTC	195
		R	ACCACGGTTCCTGAGATAACAGTC	
*EF1α*	Elongation factor-1 alpha	F	AGGCTGTCCGCAGTGTTCAAAT	178
		R	TCACACGACTGGACATACTCGTTG	
*APX*	Ascorbate peroxidase	F	TCCAACCGTTGAGTTCATCCCT	199
		R	ACCGTCAAACCCAGACCTTTCA	
*Cu/Zn SOD*	Cu/Zn Superoxide dismutase	F	TTTCCAGTCGCTCCGAATTGTCTC	186
		R	AGTCCAGTGATACGAACGTTCACC	
*MT*	Metallothionein	F	CCAGTGCAGATCAGTATCAGACCA	176
		R	CTCGTCCATCTCAGGGTACATCTT	

**Table 3:** Molecular identification, siderophore production, phosphate solubilization and minimum inhibition concentration (MIC) of the isolated bacterial and fungal strains, and the production of indoleacetic acid (IAA) by bacterial and fungi isolates in LB (Luria-Bertani) medium and 1/7 PDB (Potato Dextrose Broth), respectively, supplemented with concentrations of 0 and 250 mg·L^−1^ of arsenic.

Strains	Molecular Identification	Close Match Accession Number	MIC (mg·L^−1^)		IAA As (mg·L^−1^)		Siderophore Production †	Phosphate Solubilization ‡
			AsV	AsIII	0	250		
B4	*Pseudomonas gessardii*	MH398505.1	7000	1500	0	0	+	+
B10	*Brevundimonas intermedia*	KR811205.1	6000	1500	52.32 ± 0.9 ^a^	52.45 ± 0.3 ^a^	−	−
V7	*Fimetariella rabenhortii*	HQ406808	2500	750	40.5 ± 0.74 ^b^	41.6 ± 0.42 ^b^	−	−
V8	*Hormonema viticola*	NR137620.1	2500	720	57.64 ±1.06 ^a^	56.62 ± 2.78 ^a^	−	−

† Siderophore: (+) production, (−) no production; ‡ Phosphate solubilization (+), no solubilization (−), Different letters denote statistically significant differences (*p* < 0.05).

**Table 4:** β-glucosidase, acid phosphatase and hydrolysis of fluorescein diacetate activity (FDA) in rhizospheric soil of *T. aestivum* without the addition of As and the addition of 300 mg·Kg^−1^ of As.

	β-Gucosidase(µmoles *p*-Nitrophenol g^−1^ h^−1^)	FDA (µg Fluorescein g^−1^)	Acid Phosphatase (µmoles *p*-Nitrophenol g^−1^ h^−1^)
**Soil without As Added**			
Control	96.01 ± 8.23	12.8 ± 1.17	82.09 ± 8.01 ^a,b,c^
B4	84.39 ± 11.41	13.02 ± 1.05	90.06 ± 6.18 ^a,b,c,d^
B10	106.21 ± 13.45	14.03 ± 3.17	128.37 ± 2.88 ^d,e,f^
B4 + B0	121.35 ± 10.19	14.14 ± 1.65	123.78 ± 15.08 ^c,d,e,f^
V7	134.32 ± 4.3	15.4 ± 1.3	181.95 ± 6.53 ^g,h^
V8	97.7 ± 7.43	12.24 ± 0.93	113.37 ± 4.66 ^c,d,e,f^
V7 + V8	123.1 ± 10.1	11.02 ± 0.97	201.48 ± 10.42 ^h^
**Soil with As Added**			
Control	99.32 ± 21.27	12.15 ± 1.74	60.06 ± 5.57 ^a^
B4	109.18 ± 21	12.3 ± 0.85	70.67 ± 5.4 ^a,b^
B10	144.32 ± 18.54	13.45 ± 1.36	150 ± 4.89 ^e,f,g^
B4 + B0	114.25 ± 15.63	13.91 ± 2.77	109.72 ± 6.28 ^b,c,d,e^
V7	109.32 ± 1.4	11.68 ± 0.52	96.41 ± 7.38 ^a,b,c,d^
V8	138.51 ± 8.41	16.8 ± 1.99	158.01 ± 17.62 ^f,g,h^
V7 + V8	117.97 ± 6.75	15.75 ± 1.5	114.39 ± 8.33 ^c,d,e,f^

B4, *Pseudomonas gessardii*; B10, *Brevundimonas intermedia*; *Fimetariella rabenhortii,* V7; *Hormonema viticola* (V8). Different letters denote statistically significant differences (*p* < 0.05).

## Appendix A

**Table A1:** Arsenic content (mg·Kg^−1^) in the shoot and root of *Triticum aestivum* without the addition of As and the addition of 300 mg·Kg^−1^ of As. Control: no inoculation of microorganisms; *P. gessardii* (B4), *B. intermedia* (B10), the mixture of both (B4 + B10), *F. rabenhortii* (V7), *H. viticola* (V8), and the mixture of both (V7 + V8).

Arsenic Content (mg Kg^−1^)	Control	B4	B10	B4 + B10	V7	V8	V7 + V8
**Shoot**							
**Control**	0.37	0.51	0.41	0.6	0.52	0.45	0.42
**300 mg Kg^−1^**	8.83	6.79	6.13	6.11	7.13	6.07	6.17
**Root**							
**Control**	0.407	0.5967	0.4879	0.72	0.5772	0.495	0.4998
**300 mg Kg^−1^**	9.0949	7.9443	7.7238	7.2709	8.2708	8.0124	8.2061
